# Polycomb repressive complex’s evolutionary conserved function: the role of EZH2 status and cellular background

**DOI:** 10.1186/s13148-016-0226-1

**Published:** 2016-05-27

**Authors:** Koraljka Gall Trošelj, Renata Novak Kujundzic, Djurdjica Ugarkovic

**Affiliations:** Division of Molecular Medicine, Laboratory for Epigenomics, Rudjer Boskovic Institute, Bijenicka cesta 54, 10 000 Zagreb, Croatia; Division of Molecular Biology, Laboratory for Evolutionary Genetics, Rudjer Boskovic Institute, Bijenicka cesta 54, 10 000 Zagreb, Croatia

**Keywords:** EZH2, Polycomb repressive complexes 1–4, Evolution, Mutation, Cancer, H3K27me3

## Abstract

When assembled in multiprotein polycomb repressive complexes (PRCs), highly evolutionary conserved polycomb group (PcG) proteins epigenetically control gene activity. Although the composition of PRCs may vary considerably, it is well established that the embryonic ectoderm development (EED) 1, suppressor of zeste (SUZ) 12, and methyltransferase enhancer of zeste (EZH2)-containing complex, PRC2, which is abundant in highly proliferative cells (including cancer cells), establishes a repressive methylation mark on histone 3 (H3K27me3). From the perspective of molecular cancer pathogenesis, this effect, when directed towards a promoter of tumor suppressor genes, represents pro-tumorigenic effect. This mode of action was shown in several cancer models. However, EZH2 function extends beyond this scenario. The highly specific cellular background, related to the origin of cell and numerous external stimuli during a given time-window, may be the trigger for EZH2 interaction with other proteins, not necessarily histones. This is particularly relevant for cancer.

This review provides a critical overview of the evolutional importance of PRC and discusses several important aspects of EZH2 functioning within PRC. The review also deals with mutational studies on EZH2. Due to the existence of several protein (and messenger RNA (mRNA)) isoforms, these mutations were stratified, using the protein sequence which is considered canonical. This approach showed that there is an urgent need for the uniformed positioning of currently known EZH2 mutations (somatic—in tumors, as well as germline mutations in the Weaver’s syndrome).

Finally, we discuss EZH2 function with respect to amount of trimethylated H3K27, in a specific cellular milieu, through presenting the most recent data related to EZH2-H3K27m3 relationship in cancer. All these points are significant in considering EZH2 as a therapeutic target.

## Background

Post-translational modifications (PTMs) of histone polypeptides contribute to the regulation of gene activity through establishing a specific epigenetic regulatory network [1]. Partly due to PTMs of histones, polycomb group (PcG) proteins can control gene silencing in a considerable part of the genome but only when assembled in multiprotein polycomb repressive complexes (PRCs)—polycomb (Pc)-containing complexes (PRC1) and the enhancer of zeste-containing complexes (PRC2/PRC3/PRC4) [2, 3]. These complexes are responsible for the epigenetic memory of gene expression states and play a crucial role in the maintenance and reprogramming of cell types during normal development and during pathophysiological processes (reviewed in [4]).

### Enhancer of zeste-containing complexes during evolution

Originally identified in the fruit fly *Drosophila melanogaster* as crucial factors in maintaining the repressed state of developmental regulators such as homebox *HOX* genes [5], the Pc-group proteins were shown to be highly evolutionary conserved [6]. For example, PRC2 is detected even in unicellular eukaryotes, alga *Chlamydomonas* [7] and yeast *Cryptococcus neoformans* [8].

The widespread presence of PRC2, from unicellular organisms to humans, points out its significance for preserving a specific module(s) of gene repression. Evolutionary processes have offered unique ways of PRC2 composing (Table 1): (1) *Drosophila* contains four core proteins: enhancer of zeste E(Z); suppressor of zeste 12 SU(Z)12; extra sex combs (ESC) and the histone binding protein p55. The E(Z) protein contains a SET domain which exerts histone lysine methyltransferase activity (KMT), able to catalytically add up to three methyl groups at the target lysine residue K27 of histone 3 (H3). The E(Z) possesses the SANT domains involved in histone binding and a C5 domain required for interacting with SU(Z)12 [9]; (2) Yeast *Cryptococcus neoformans* PRC2 has no homolog of SU(Z)12 but contains two additional proteins, Bnd1 and Cc1, specific for this species [8]; (3) In nematode *Caenorhabditis elegans*, only homologs of E(Z) and ESC are found, MES-2 and MES6, respectively. These two proteins make a PRC2 together with a MES-3 protein which has no homolog in any other model organism, and such complex is involved in X-chromosome repression [10]; (4) Plants such as *Arabidopsis thaliana*, due to gene duplications, have three homologs of E(Z): CLF, MEA, SWN; three homologs of SU(Z)12: FIS, VRN2, EMF2; and five homologs of p55: MSI1-5, while only one homolog of ESC is present (reviewed in [11]). The combinations of these proteins make at least three distinct PRC2 which are involved in different developmental processes. FIS-PRC2 is similar to its mammalian counterpart and regulates expression of imprinted genes and cell proliferation. EMP-PRC2 acts like *Drosophila* and mammalian PRC2 in maintaining the repressed state of homeotic genes and, together with the third complex, VNR-PRC2, regulates flower time [11] .

**Table 1:** PRC2 core proteins in model organisms [4, 8]

Yeast *Crytococcus neoformans*	Nematode *Caenorhabditis elegans*	Plant *Arabidopsis thaliana*	Fly *Drosophila melanogaster*	Mouse and human *Mus musculus* and *Homo sapiens*
Bnd1, Cc1				
	MES-3			
EED1	MES-6	FIE	ESC	EED
EZH2	MES-2	CLF	E(Z)	EZH1
		MEA		EZH2
		SWN		
		FIS2	SU(Z)12	SU(Z)12
		VRN2		
		EMF2		
MSI1		MSI1-5	p55	RbAp46/48

The duplication of E(Z) gene resulted in two mammalian E(Z) proteins, EZH1 and EZH2 (Table 1), as well as two PRC2 complexes, each containing one of these two EZH-proteins. Accordingly, mammalian PRC2 is composed of four core subunits: EZH1/EZH2, SUZ12, embryonic ectoderm development (EED), and retinoblastoma(Rb)-associated protein 46/48 (RbAp46/48).

Although present in similar PRC2 complexes and controlling an overlapping set of genes, EZH1 and EZH2 are considerably different. PRC2-EZH2, abundant in highly proliferative cells, establishes a repressive H3K27me3 mark on PRC2 target genes. PRC2-EZH1, which is abundant in non-dividing cells, likely restores this repressive mark, either as a result of its disappearance due to demethylation or by histone exchange [12].

### PRC composition is flexible and cell-type specific

H3K27 is not the only histone-related substrate for EZH2, as the PRC-partners may direct the EZH2 to other substrates. For example, an EED isoform 2 (Eed2) and NAD-dependent histone deacetylase Sirt1 specifically associate within the PRC4 which is needed for methylating linker histone H1 (H1K26) [13]. This modification is specific for cancer and undifferentiated embryonic stem (ES) cells.

**Fig. 1:**
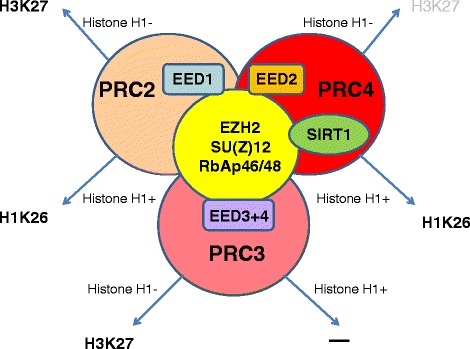
Association of PRC-EZH2 complexes with different EED isoforms in the presence (H1+) or absence (H1−) of linker histone H1 directs EZH2-mediated methylation towards H3K27 or H1K26. PRC2, which contains the longest form of EED (EED1), is able to methylate isolated histone H3. When targeted to oligonucleosomes containing linker histone H1, PRC2 methylates histone H1 rather than histone H3. PRC3, containing EED3 and EED4, methylates nucleosomal histone H3, but its methyltransferase activity is inhibited by histone H1. PRC4, containing EED2 and NAD-dependent deacetylase SIRT1, methylates histone H1 when present, but has also low methylating capacity towards H3K27 in the absence of histone H1 (depicted in gray) [13, 14]

There is a whole spectrum of variations relating to the dynamic exchange of protein partners (AEBP2, Pcl1/2/3 (PHF1/MTF2/Pcl3t), Jarid2) which may be temporary members of PRC2. This “exchange phenomenon” should not be surprising, as the specific biological effect mediated by PRCs—broad control of gene activity must be achieved very precisely, in a cell-type specific manner and during a controlled time-window (Fig. 1) [14]. For example, Jarid2—a member of Jumonji family of histone demethylases without enzymatic activity—was identified as a part of PRC2, in interaction with Ezh2. Jarid2 binds DNA with a slight preference for GC rich sequences [15] and recruits PcG proteins to target genes [16].

Although there are several possibilities related to flexible ways of composing the content of PRC2 (as discussed), it is known that the minimum components required for methyltransferase activity of the PRC2/EED-EZH2 complex are EED, EZH2, and SUZ12. The coordinated activity of these proteins is essential for establishing di- and tri-methylated H3K27 (H3K27me2/me3) marks which are associated with facultative heterochromatin. These marks present the hallmark histone modification produced by Ezh1 and Ezh2 activity within the PRC2. However, the precise mechanism that governs PRC2 recruitment to chromatin in mammals still needs to be defined.

Recognizing PRC2 functioning as a holoenzyme whose components act together to establish interaction with chromatin in a stepwise manner, Margueron and Reinberg [17] have proposed the following several-steps model: (a) interaction of Jarid2 and AEBP2 with DNA [18, 19]; (b) interaction of RbAp46/48 with histones H3 and H4 [20]; (c) interaction of Eed with H3K27me3 [21]; (d) interaction of Plcs with an unknown histone mark; and (e) interaction of PRC2 subunits with long non- coding RNA (lnc RNA).

These molecular events are highly conserved. In mammals (reviewed in [22]) are well documented through the interaction of lnc RNA X inactive-specific transcript (Xist) with EZH2 and the consequential recruitment of PRC2 to the X-chromosome leading to its inactivation. In plants, cold induced lnc RNA COLDAIR interacts with plant E(Z) homolog CLF [23] and recruits PRC2 to the target locus in a way similar to the Xist in mammals. In malignant neoplasms, as shown in gastric cancer, overexpressed lnc RNA 00152 needs to bind to EZH2 in order to exert oncogenic potential through recruiting the PRC2 to promoters of tumor suppressor (TS) genes p15 and p21 [24].

Learning about the evolutional significance of PRC2 in the control of cellular proliferation and differentiation is very important for understanding some basic pathophysiological processes. For example, plants with double mutation of two out of three E(Z) homologs, clf and swn, undergo normal seed development, but produce a mass of proliferating, undifferentiated tissue resembling cancer, instead of a differentiated shoot after germination [25].

### Several aspects of aberrant EZH2 function in cancer

In humans, the EZH2 mutation may occur in a germline, resulting in clinical features known as the Weaver syndrome, originally described in 1974 [26]. In 2011, mutational analysis of EZH2 in 48 Weaver syndrome patients revealed 44 missense and four truncated mutations. All but two SET domain mutations (R684C and S652C), which were present in five and two unrelated individuals, respectively, were distributed throughout the gene, without specific clustering [27]. Only two germline EZH2 mutation-positive individuals developed hematological malignancies: E745K (a lymphoma diagnosed at the age of 13) and an A682T mutation (acute lymphoblastic leukemia (ALL) and neuroblastoma developed at 13 months).

In 1996, EZH2 was first discovered as a binding partner of Vav oncoprotein in hematological malignancies [28]. These neoplasms were, in addition to breast and prostate cancer, pioneering models for investigating the function and role of EZH2. Its overexpression was first associated with amplification at 7q35 (more than four *EZH*2 copies per cell) in approximately 15 % of the 225 analyzed breast cancers (BCs) [29]. In 2010, EZH2 point mutation (Y641) in SET domain was first found in 7 % of large follicular lymphomas and 22 % of diffuse B cell lymphomas [30]. It was also found in approximately 3 % of melanomas [31]. The discovery of two additional SET domain mutations (A677G and A687V) followed [32, 33].

These “gain of (methyltransferase) function” mutations are responsible for the oncogenic mode of EZH2 action. Contrary to wild-type (WT) EZH2, which loses activity when progressively more methyl groups are incorporated into H3K27, all tested Y641 mutant enzymes (Y641F/N/S/H/C) displayed the opposite trend (H3K27me0:me1:me2 kcat/Km ratio: 13:4:1 (WT) vs 1:2:22 (Y641) [34]. Since one cell possesses both wild- and mutant types of the EZH2 allele, there appears to be dependency on the coordinated activity of both alleles.

Aberrant activity of PRC2 can result from aberrant EZH2 expression, without chromosomal amplification, as a consequence of diverse aberrations which are present in cancer cells. For example, comprehensive analyses of transcriptome and epigenome data obtained from adult T cell leukemia (ATL) cell lines, normal CD4^+^T cells, human T-lymphotropic virus type 1 (HTLV-1)-immortalized and transformed T cells show the importance of increased, NF-κB dependent expression of EZH2 (both RelA and RelB were shown to be bound to EZH2 promoter) which further activates NF-κB through silencing of microRNA (miR)-31. Of interest for this model, H3K27me3 was enriched in the promoter of transcriptionally downregulated H3K27me3 demethylase KDM6B (JMJD3), which also may compromise the balance between epigenetic “writers” and “erasers.” It was shown that HTLV-1 protein Tax binds to EZH2, without affecting the PRC2 composition. As a result, the pattern of H3K27me3 accumulation significantly overlaps in ATL- and HTLV-1-immortalized cells. Since HTLV-1 infected cells are sensitive to EZH2 inhibition, this research data may be a ground for introducing EZH2 inhibitors for treating asymptomatic, HTLV-1 infected individuals [35].

Hepatitis B virus (HBV)-associated hepatocellular cancer (HCC) represents another interesting model for studying the abberant expression of tumor suppresive miRs in respect to PRC2 activity in a setting of prolonged viral infection. In the HBV-HCC model, co-expression of transcription factor (TF) YY1 and EZH2 are associated with silencing several, multiple YY1 binding sites-containing suppressive miRs and relate to short disease-free survival [36]. YY1 can interact with both EZH2 and SUZ12 [37] and recruits the PRC2 complex to chromatin. The discovery of this oncogenic mechanism, which was responsible for silencing of five highly NF-κB suppresive miRs, pointed out the importance of coordinated action of YY1 and EZH2 for focal reshaping of chromatin.

The already mentioned tumor suppressor miR-31 was shown to be silenced in prostate cancer cells through presence of H3K27me3 on its promoter [38]. The absence of miR-31 in t(4;14) positive multiple myeloma (MM) patients (15–20 %) allows for pro-oncogenic activity of its target—multiple myeloma set domain methyltransferase (MMSET), which establishes histone mark H3K36me2 and induces a global reduction H3K27me3 [39]. However, in this scenario, specific loci exhibit enhanced recruitment of EZH2, leading to misregulation of specific polycomb target genes.

It was recently shown that H3K27me3 enriched genes in experimental models of MM significantly overlap with underexpressed genes in MM patients with poor survival [40]. Of interest, although applying EZH2 inhibitor, E7438 induces reproducible re-expression of crucial epithelial tumor suppressor genes (including *CDH*1) in 13 tested MM cell lines, there are many questions arising from a high variability in E7438 sensitivity in the proliferation assays [41].

All these examples show that there are many factors that may influence EZH2 and are influenced by EZH2. Accordingly, EZH2 pharmacological inhibition may have various effects.

In addition to “gain of function” mutations, there are also EZH2 “loss of function” mutations discovered in hematological malignancies originating from myeloid cells, commonly joined with unipaternal disomy (UPD) [42]. The proposed model of EZH2 “loss of function” mutations (of which the majority were found in the SET domain) attributes their contribution to be forming cancer stem cells, via HOXA9 mediated self-renewal of myeloid progenitors. A complex in vivo model (transplantation of bone marrow (BM) cells from 8–12-week-old Cre-ERT;Ezh2fl/fl CD45.2 mice into lethally irradiated CD45.1 recipient mice and deletion of Ezh2 at 6 to 8 weeks posttransplantation) reveals that complete lack of EZH2 activity in hematopoietic stem cells (HSCs) predisposed mice to heterogenous malignancies (MDS, MDS/MPN, MDS/MPN associated with trombocytosis, and T cell acute lymphoblastic leukemia). The same experimental model showed locus-specific repositioning of EZH1 to EZH2 targets (3605 genes in contrast to 969 “EZH2 targets only”) and its ability to re-repress them during prolonged period of time (9 months) [43]. All these data clearly indicate that EZH2 function, in both physiological process and in various pathogenic events, must be studied in a broad context, keeping in mind that its binding partners contribute to specificity of its functioning, in a particular cellular setting.

### Which mutation is “the right one”?

**Fig. 2:**
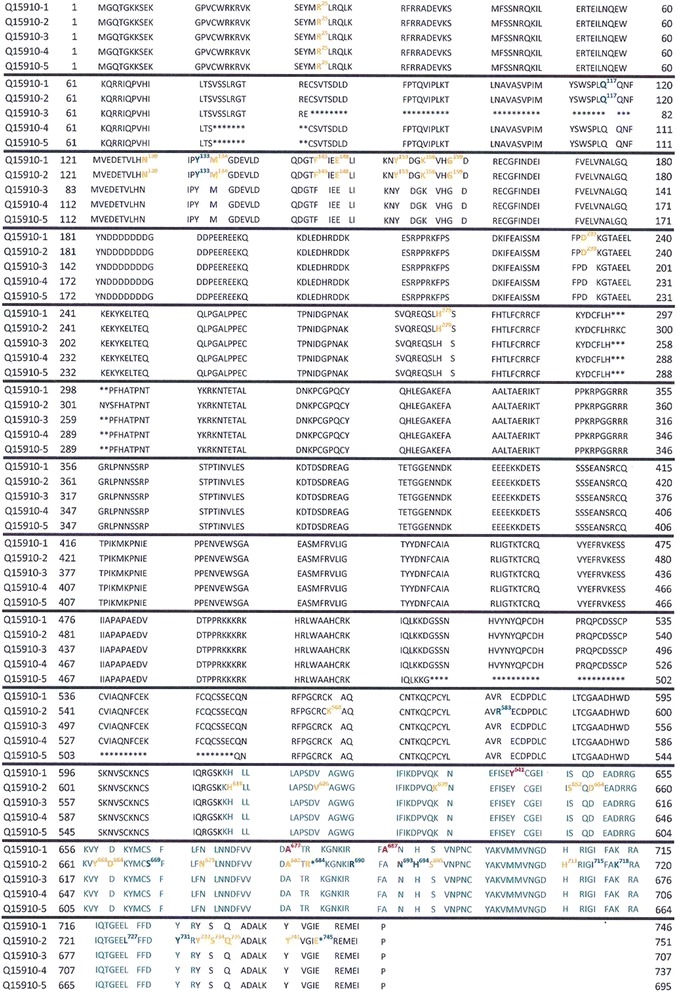
Alignment of five EZH2 isoforms protein sequences (UniProt). SET domain is shown in *green* (Q15910-1 AA 612–727; Q15910-2 AA 617–732; Q15910-3 AA 573–688; Q15910-4 AA 603–718; Q15910-5 AA 561–676). Germline mutations [27] are shown in *orange*, “loss of function” mutations [42] in *blue*, and “gain of function” mutations [26, 30, 32, 33, 44] in *red*. All mutations listed in the cited references are marked on respective isoform sequences, highlighting the lack of uniformity in annotating mutations according to consensus sequence (Q15910-1). Therefore, mutation A677 (in isoform 1) is listed as somatic, activating mutation and at the same time, annotated as mutation A682 (in isoform 2), has been listed as germline mutation which was discovered in the Weaver syndrome patient who developed ALL and neuroblastoma in early childhood. This is in accord with the oncogenic potential of this mutation. Inactivating mutations R684 in isoform 2 (corresponding to R679 in isoform 1) and E745 in isoform 2 (corresponding to E740 in isoform 1) have been shown to be mutated in Weaver syndrome patients. None of the five patients with inherited mutation R684C (present as somatic mutation in one 82-year-old patient suffering from chronic myelomonocytic leukemia) developed malignant disease at the time of testing for germline mutation of EZH2. Germline mutation E745K (isoform 2) was present in a patient who developed non-Hodgkins lymphoma at the age of 13. Somatic mutation of this codon was detected in one patient with chronic myeloic leukemia during blast crisis.

The problem that occurs when comparing the results of EZH2 mutational analyses coming from different sources relates to amino acids positioning in the EZH2 sequence. For example, “gain of function” mutations are listed according to the protein sequence that is considered “canonical” (UniProtBD/Swiss-Prot Q 15910–1; 746 amino acids (AA)) [30, 32, 33]. On the other hand, “loss of function” [34], and germline mutations [27], were positioned according to the longest protein isoform of EZH2 (UniProtBD/Swiss- Prot Q 15910-2; 751 AA). The absence of uniformity may be confusing. For example, the already mentioned inherited mutation discovered in the Weaver syndrome patient suffering from ALL (A682T) [27] corresponds to alanine 677 mutation (A677G) in B cell lymphoma [30]. Similarly, a rare EZH2 breast cancer mutation described as A692V [44] corresponds to B cell lymphoma mutation at position 687 [33]. The difference of five amino acids corresponds to the difference between Q15910-1 and Q15910-2 isoforms (HP → HRKCNYS), which are identical in the first 297 amino acids (Fig. 2). The basic data on currently known EZH2 protein isoforms and their coding messenger RNAs (mRNAs) are presented in Table 2. The hope is that future presentations of EZH2 isoforms and the positions of mutated codons will be done in a more uniform manner.

**Table 2:** Human EZH2: five proteins and corresponding mRNA splice variants are currently deposited. Although mutational analyses of EZH2 refer to the ordinal number of mutated amino acids, they rarely identify the isoform which is the basis for numbering them

UniProtBD/Swiss-Prot	Transcript variants (TV)	mRNA NCBI	mRNA length (nt)	Coding region	cDNA length (nt)	Protein size (AA)
15910-1	TV3	NM_001203247.1	2708	194-2434	2241	746
15910-2	TV1	NM_004456.4	2723	194-2449	2256	751
15910-3	TV2	NM_152998.2	2591	194-2317	2124	707
15910-4	TV4	NM_001203248.1	2681	194-2407	2214	737
15910-5	TV5	NM_001203249.1	2682	321-2408	2088	695

Stratified presentation of mutations published in previous studies [27, 30–34, 44] reveals identical type/position of three germline (G) and three somatic (S) mutations. One mutation was reported as oncogenic (lymphoma; S&G:A677T), and two other ones were reported as suppressive (S:E741fs → G:E741K; S:R679C/P → G:R679C/H) (Fig. 2; UniProtBD/Swiss- Prot Q 15910-1). It remains to be seen whether any of these EZH2 mutations act as a “Janus” mutation in the RET protooncogene (germline mutation that acts simultaneously as both a gain-of-function and a loss-of-function mutation) [45].

Data related to the functional differences of EZH2 splice variants is scarce. The expression level of EZH2 transcript variants 1 and 3 was shown to be similar in 22 tested human tissue samples. Forced expression of corresponding protein isoforms (Q 15910-2 and Q 15910-3; Table 2) in pancreatic cancer cells revealed that each protein isoform has an affinity for a preferential gene cluster (36.3 and 47.6 % genes were repressed by EZH2β (Q 15910-3) and EZH2α (Q 15910-2), respectively, while repression of remaining 16.1 % genes needed the presence of both isoforms) [46]. The data indicates that the different EZH2 cell-specific mRNAs, and protein isoforms may have functional importance, including for the clinic, as already shown for some other genes [47, 48].

### Specific cellular background and multiple roles of EZH2

EZH2 binding affinity for both histones and non-histone substrates may partially explain why targeted silencing of EZH2 leads to bidirectional change of gene expression, in a specific cellular context-dependent manner [49]. Some examples are: (1) EZH2 binds to RelA/RelB in BC cells and regulates the NF-κB target genes in a positive (*IL*-6, *TNF*) or negative way, depending on estrogen receptor (ER) status rather than the EZH2 histone methyltransferase activity. In ER+ BC cells, ER recruits PRC2 for enforcing a repressive chromatin modification at NF-κB target genes. (2) In squamous cell carcinomas (SCC), EZH2, through repressing IκB kinase α (*IKK*1) promoter, leads to IKK1 silencing [50]. In any other types of tumor, this would be a suppressive effect. However, it is oncogenic in SCCs because IκB kinase α has a tumor suppressive role in these tumors [51]*.* 3. Finally, it seems that EZH2 catalytical activity does not have the most significant role for an increased rate of growth in some SWI/SNF-mutant cancers [52]. Instead, the stabilization of PRC2, dependent on EZH2 threonine 487 (T487) phosphorylation (Prot Q 15910-1), seems to be essential, at least in this particular scenario [53]. However, this phenomenon may be abrogated by presence of mutant K-ras.

In non-small cell lung cancer (NSCLC), the type of substitution at 12th codon of K-ras determines activation of a specific pro-proliferative signaling pathway. Cells with K-RAS ^G12D/+^ or K-RAS ^G12C/+^ have primarily activated PI3/AKT and MEK/ERK signaling pathways, respectively [54]. Accordingly, activation of EZH2, which was shown to be dependent on K-ras mutants, may be inhibited by specific inhibitors of mutation-type dependent downstream signals. This is important because one of significant pro-oncogenic activities of EZH2 depends on activated AKT which, through EZH2, phosphorylates and activates oncogenic STAT3 [55] .

A generation of mice with Cre-recombinase-activated conditional oncogenic K-ras allele (K-ras ^G12D/+^), along with either mild Ezh2 overexpression (Ezh2^LSL^) or lost PRC2 function achieved by conditional deletion of Eed1 (Eed^fl/fl^), joined with conditional deletion of p53 (Trp53^fl/fl^), revealed that the genotype K-ras^G12D/+^; Trp53^fl/fl^; and Eed^fl/fl^ develops the most aggressive, mucinous NSCLC. In this genetic setting, which is relevant for human pathology (mutations of K-RAS and P53 are present in 35 and 40 % NSCLCs, respectively), Eed1 acts as a tumor suppressor gene. In the presence of WTp53, Kras^G12D/+^;Eed^fl/fl^ mice developed NSCLs which were, although smaller than Kras^G12D/+^ /Ezh2^LSL^ tumors, characterized by life incompatible inflammation in alvelolar spaces. In vitro, the inhibition of EZH2, achieved through the prolonged exposure of human K-RAS-mutant NSCLC cells to an inhibitor of EZH2 catalytic activity (GSK126), resulted in a strong increase of inflammatory genes (i.e., *IL-6*) associated with microenvironment-regulated tumor progression. Based on these and many more results coming from the cited study [56], it was suggested that PRC2 can hold opposing functions, depending on the stage of tumor development and the genetic make-up of the tumors (as presented here), with respect to p53 status. Accordingly, this and other studies clearly show a rationale for the combined application of PRC2 inhibitors and anti-inflammatory drugs. In the model of hematopoietic stem cells, EZH2 loss was recently shown to result in the expression of fetal gene signature, including upregulation of fetal-specific Lin28b which encodes RNA-binding protein that prevents maturation of miR-let-7 which is specific for adult HSCs. Activation of fetal gene signature in EZH2-deficient adult bone marrow HSCs was shown to result in fetal-like high self-renewal capacity and increased propensity to undergo malignant transformation [57]. Enforced expression of Lin28b has been reported to impair T cell development in vivo, leading to developing an aggressive peripheral T cell lymphoma, accompanied by a decrease in let-7 expression, surge of IL-6, activation of NF-κB, and infiltration of B cells leading to an inflammatory microenvironment [58].

The proper anti-tumor function of T cells depends on the differentiation of naive and memory T cells into effector cells. Metabolic switch from oxidative phosphorylation to aerobic glycolysis is mandatory for T cell activation. Highly glycolytic ovarian cancer cells were recently shown to impose glucose restriction on tumor-infiltrating T cells, thereby inhibiting this metabolic switch. Low glucose availability results in upregulation of EZH2-targeting miR-26a and miR-101 with subsequent EZH2 downregulation. This is consequential for T cell effector function, since EZH2 activates the Notch pathway that stimulates T cell polyfunctional cytokine expression and their survival, which was shown to be impaired in many tumors. These results point to the different effects that systemic inhibition of EZH2 may have on tumor cells and T cells, warranting special caution when considering such epigenetic intervention [59].

### H3K27me3 as a measure of EZH2 activity

There are many EZH2-related scenarios and none of them is simple. When analyzed in five well-defined subtypes of BC, the highest EZH2 expression, joined with a very low level of H3K27me3, was found in basal-like, triple negative BC [60], known for its distinctly aggressive nature [61]. This inverted pattern (EZH2↑, H3K27me3↓), further confirmed in a basal-like BC cell lines, represents the negative prognostic marker in BC patients [60, 62]. There are a few studies in which a decreased level of H3K27me3 was associated with a poor outcome in different malignant tumors (breast, ovary, pancreas, lung) [63, 64]. These results, together with those showing that solid tumors (prostate, breast) can develop even in the absence of Ezh2 [44], challenge the strength of EZH2 as the epigenetic driver of oncogenesis [65], at least in the stated tumor types. This data, supported by a broad analysis of human transcriptome data sets (131 prostate cancers (plus 19 metastases), 146 BCs) indicates that EZH2 expression “follows” the rate of cellular division, is under control of proliferation cues, and “passively” correlates with proliferation and proliferation markers (primarily Ki-67), in order to maintain the cellular level of H3K27me3.

It was suggested that EZH2 overexpression should be considered from two perspectives: (a) through coupling its expression to proliferation and (b) coupling it to proliferation-independent, amplification-related, copy number-driven, expression [44].

However, this approach should be considered in a specific cellular milieu and should not be applied non-selectively, to all types of malignant tumors:

In many systems, EZH2 supports stem cell maintenance by repressing differentiation. But, in neural crest stem cells (NCSCs), which are the source of melanocytes, it specifically promotes the acquisition of a mesenchymal fate [66]. EZH2 is essential for melanoma initiation and growth, during which EZH2 and Ki-67 positive cells significantly correlate, just like in the BC model. Increased expression of EZH2 in melanoma strongly correlates with shorter overall survival (OS) and earlier development of distant metastases [67, 68]. EZH2-mediated repression of the tumor suppressor adenosylmethionine decarboxylase 1 (AMD1) appears to be of the greatest importance for these processes. The role of this gene, as well as its repressor, EZH2, needs to be further investigated and validated.

## Conclusions

The function of any biomolecule must be considered in a specific cellular setting. Accordingly, cell-type specific signals which constantly change during adaptive responses to various stimuli are the basis of an epigenomic dynamic network, reflecting both—the type of the cell and the type of the stimulus in a given time-window. Understanding the exact role of EZH2 in such a complex system is not an easy task. There is convincing mechanicistic data confirming the oncogenic function of EZH2 related to PRC2 functioning (repression of tumor suppressor genes through H3K27me3) in several biological models. Numerous studies, however, interpret an increased EZH2 immunoreactivity score as an unquestionable oncogenic event. The fact that the score does not necessarily reflect the presence of a functional PRC2 and/or its increased recruitment to chromatin seems to be all too often neglected.

When considering EZH2 as a therapeutic agent, one must take into account these parameters, together with an understanding of the functional consequences of EZH2 mutations and the cancer patient’s specific cellular oncometabolome with respect to systemic inflammatory reactions.

While the targeted inhibition of EZH2 catalytic activity emerges as a promising therapeutic intervention, it still has many other cellular-specific functions which must be carefully evaluated to avoid broad side effects. These issues are further discussed in a broader context elsewhere in this issue of Clinical Epigenetics [69].

## Abbreviations

AA: amino acid
ALL: acute lymphoblastic leukemia
AMD1: adenosylmethionine decarboxylase 1
ATL: adult T cell leukemia
BM: bone marrow
BC: breast cancer
E(Z): enhancer of zeste
EED: embryonic ectoderm development
ER: estrogen receptor
ES cells: embryonic stem cells
ESC: extra sex combs
EZH2: enhancer of zeste homolog 2
G: germline
H3K27: lysine 27 of histone 3
HBV: hepatitis B virus
HOX: homebox
HSC: hematopoietic stem cell
HTLV-1: human T-lymphotropic virus type 1
*IKK*1: IκB kinase α
*IL-6*: interleukin-6
KMT: lysine methyltransferase
lnc RNA: long non-coding RNA
MDS: myelodisplastic syndrome
miR: microRNA
MM: multiple myeloma
MMSET: multiple myeloma set domain methyltransferase
MPN: myeloproliferative neoplasms
NCSCs: neural crest stem cells
NSCLC: non-small cell lung cancer
OS: overall survival
PcG: polycomb group
PRCs: polycomb repressive complexes
PTMs: post-translational modifications
RbAp46/48: retinoblastoma(Rb)-associated protein 46/48
S: somatic
SCC: squamous cell carcinoma
SU(Z)12: suppressor of zeste 12
TF: transcription factor
TS: tumor suppressor
UPD: unipaternal disomy
WT: wild-type
Xist: X inactive-specific transcript
